# Reliability of artificial intelligence-driven markerless motion capture in gait analyses of healthy adults

**DOI:** 10.1371/journal.pone.0316119

**Published:** 2025-01-22

**Authors:** Brandon Schoenwether, Zachary Ripic, Mitchell Nienhuis, Joseph F. Signorile, Thomas M. Best, Moataz Eltoukhy

**Affiliations:** 1 Department of Kinesiology and Sport Sciences, University of Miami, Coral Gables, FL, United States of America; 2 Department of Orthopaedics, University of Miami Health System—Sports Medicine Institute, Coral Gables, FL, United States of America; 3 Miller School of Medicine, University of Miami, Miami, FL, United States of America; 4 Department of Industrial and Systems Engineering, University of Miami, Coral Gables, FL, United States of America; 5 Department of Physical Therapy, University of Miami Miller School of Medicine, Miami, FL, United States of America; 6 Center on Aging, University of Miami Miller School of Medicine, Miami, FL, United States of America; La Trobe University, AUSTRALIA

## Abstract

The KinaTrax markerless motion capture system, used extensively in the analysis of baseball pitching and hitting, is currently being adapted for use in clinical biomechanics. In clinical and laboratory environments, repeatability is inherent to the quality of any diagnostic tool. The KinaTrax system was assessed on within- and between-session reliability for gait kinematic and spatiotemporal parameters in healthy adults. Nine subjects contributed five trials per session over three sessions to yield 135 unique trials. Each trial was comprised of a single bilateral gait cycle. Ten spatiotemporal parameters for each session were calculated and compared using the intraclass correlation coefficient (ICC), Standard Error of the Measurement (SEM), and minimal detectable change (MDC). In addition, seven kinematic waveforms were assessed from each session and compared using the coefficient of multiple determination (CMD). ICCs for between-session spatiotemporal parameters were lowest for left step time (0.896) and left cadence (0.894). SEMs were 0.018 (s) and 3.593 (steps/min) while MDCs were 0.050 (s) and 9.958 (steps/min). Between-session average CMDs for joint angles were large (0.969) in the sagittal plane, medium (0.554) in the frontal plane, and medium (0.327) in the transverse plane while average CMDs for segment angles were large (0.860), large (0.651), and medium (0.561), respectively. KinaTrax markerless motion capture system provides reliable spatiotemporal measures within and between sessions accompanied by reliable kinematic measures in the sagittal and frontal plane. Considerable strides are necessary to improve methodological comparisons, however, markerless motion capture poses a reliable application for gait analysis within healthy individuals.

## Introduction

Gait analysis, the examination of the kinetic, kinematic, and spatiotemporal elements that comprise walking, is utilized in fields such as engineering [1], physical therapy [2], and sports medicine [3]. Gait analysis tools are used to provide measures for both predictive and preventative use in various populations including healthy adults [4], Parkinson’s Disease (PD) patients [5], and athletes [6]. The most widely accepted procedure for gait analysis is three-dimensional (3D) marker-based motion capture using optoelectronic cameras paired with passive reflective markers located on key anatomical landmarks. These markers are placed with high precision, allowing accurate 3D reconstruction of the skeletal system. The virtual skeletal model is then used for the calculation of the kinetic, kinematic, and spatiotemporal components in gait analyses.

Traditionally, gait analysis has been performed in a laboratory environment, necessitating a large operating space and highly controlled settings, reducing accessibility to many clinicians. Furthermore, the expertise and time commitment required to capture and process data, as well as inherent problems like soft tissue artifact [7, 8] and inability of cameras to detect specific markers [9] present additional obstacles to marker-based system use in clinical and athletic environments. Alternatives to laboratory-based motion capture have emerged through the advancement of hardware development and artificial intelligence (AI). By employing AI paired with high-speed videos, clinicians have a new opportunity to conduct gait analyses with minimal environmental or technological barriers. Markerless motion capture (MMC) optimizes patient visits by leveraging AI with residual deep learning networks, which can handle a vast quantity of image and video data [10]. Such advancements have driven innovations in 3D human pose estimation (3D-HPE) by training the AI networks for keypoint recognition, allowing them to extract salient features and generate mappings of body parts within images [11]. AI-based MMC systems eliminate marker-related errors, reduce data collection times, and eliminate the need for specialized clothing and environments, providing substantial benefits in clinical and athletic settings. These combined attributes make AI-based MMC systems a practical and accessible choice for gait analysis.

Currently, research comparing gait data from traditional marker-based motion capture to MMC in healthy adults has demonstrated excellent validity across spatiotemporal [12, 13] and kinematic variables [14, 15]. Increased use of MMC can be partially attributed to innovations in 3D-HPE techniques, allowing for greater precision when estimating joint centers [16], increased accessibility to motion capture tools [17], and accelerated workflows that reduce processing times [18]. However, as the use of computer vision is a recent development, the reliability of kinematic and spatiotemporal MMC gait data in healthy adults remains limited [19]. The opportunities afforded by MMC systems must be approached cautiously as there are many considerations associated with choosing a MMC system. These include feasibility based on the type and number of cameras [20], ease of use dependent on automated data processing [21], and accurate representation of 3D motion reliant on the quality of image data input from keypoint annotations when developing the 3D-HPE model [13, 15, 19]. The previous work highlighted above has assessed the advantages and limitations of these MMC systems during gait analysis, the primary use-case for each system. Alternatively, the efficacy of these systems to handle sport-specific movements has been confined to laboratory settings, as opposed to live sporting-events [22–25]. Conversely, the generalizability of domain specific systems such as KinaTrax, designed for accurate representation of 3D sport-specific movements, must continue to be examined before it can be implemented in gait analysis. Given the efficient and user-friendly nature of MMC to analyze gait, its prominence in clinical settings is certain to grow, and therefore, the reliability of specific systems must be established.

Therefore, the purpose of this study was to assess the within- and between-session reliability of kinematic and spatiotemporal gait results from the KinaTrax system during three sessions of the AI-based MMC data collection in healthy adults. We hypothesized two main findings from this work: 1) for the MMC system, there would be good to excellent agreement in the sagittal, frontal, and transverse planes for all gait kinematics among trials in a single session and for average gait values between sessions, except for comparisons of the hip joint and pelvis segments, which show moderate agreement in the frontal and transverse planes based on previous work [14]. 2) There would be excellent within- and between-session agreement for spatiotemporal parameters when using the MMC system, as demonstrated previously by Ripic et al. [12].

## Materials and methods

### Participants

Nine participants were recruited from the University community for this study. Subjects were recreationally active individuals (M/F: 5/4; age = 21.44 ± 1.94 years; mass = 72.98 ± 15.41 kg; height = 1.74 ± 0.11 m), who reported no musculoskeletal impediments or neurodegenerative diseases that would impair gait function. Sample size justification is highlighted by previous work in which the reliability of a commercially available motion capture system was examined for gait measures across three sessions. Analysis of standard, marker-based systems utilizing a single laboratory setup such as Manca et al. assessed two individuals [26], while Kaufman et al., operating across three different laboratories, and three different motion capture systems assessed ten individuals [27]. Emerging MMC systems have also assessed sample sizes kindred to this study, when Kanko et al. (2021) enrolled eight healthy adults across three sessions [19].

### Testing procedures

This study was approved by the University’s Institutional Review Board (#20201216, 30/10/2020) with data collection commencing 01/04/2022 and concluding 01/12/2022. Subjects were brought into the laboratory on three separate occasions separated by seven days, with each session lasting approximately 30 minutes. At the first session, the testing protocol along with the benefits and risks of the study were explained, after which, written informed consent was obtained. Following informed consent, subjects were familiarized with the testing environment and procedure, and anthropometric data were measured. This protocol overlapped with another protocol that sought to compare the results between KinaTrax and a commercially available marker-based system [28]. Therefore, the first session had procedures in which participants were measured concurrently while in the final two sessions, participants were measured exclusively with KinaTrax. While it is possible the presence of markers in the first session may alter the joint tracking of the 3D-HPE model, to our knowledge the proprietary model training dataset is void of any training data that incorporates individuals wearing retroreflective markers. An important distinction is that the markers are placed on known anatomical landmarks and then used to derive joint centers in post-processing while the 3D-HPE model is trained to locate the joint center directly.

Using a 10-m walkway (2m approach, 3m collection area, 5m deceleration), gait trials were conducted in a single direction, adhering to the standard protocol of the laboratory. Data were collected using an eight-camera markerless motion capture system (KinaTrax Inc., Boca Raton, FL) recording at 100 frames per second. As opposed to standard marker-based calibrations, the markerless system collects a single background image of the capture volume that is used for laboratory configuration and post-processing.

**Fig 1:**
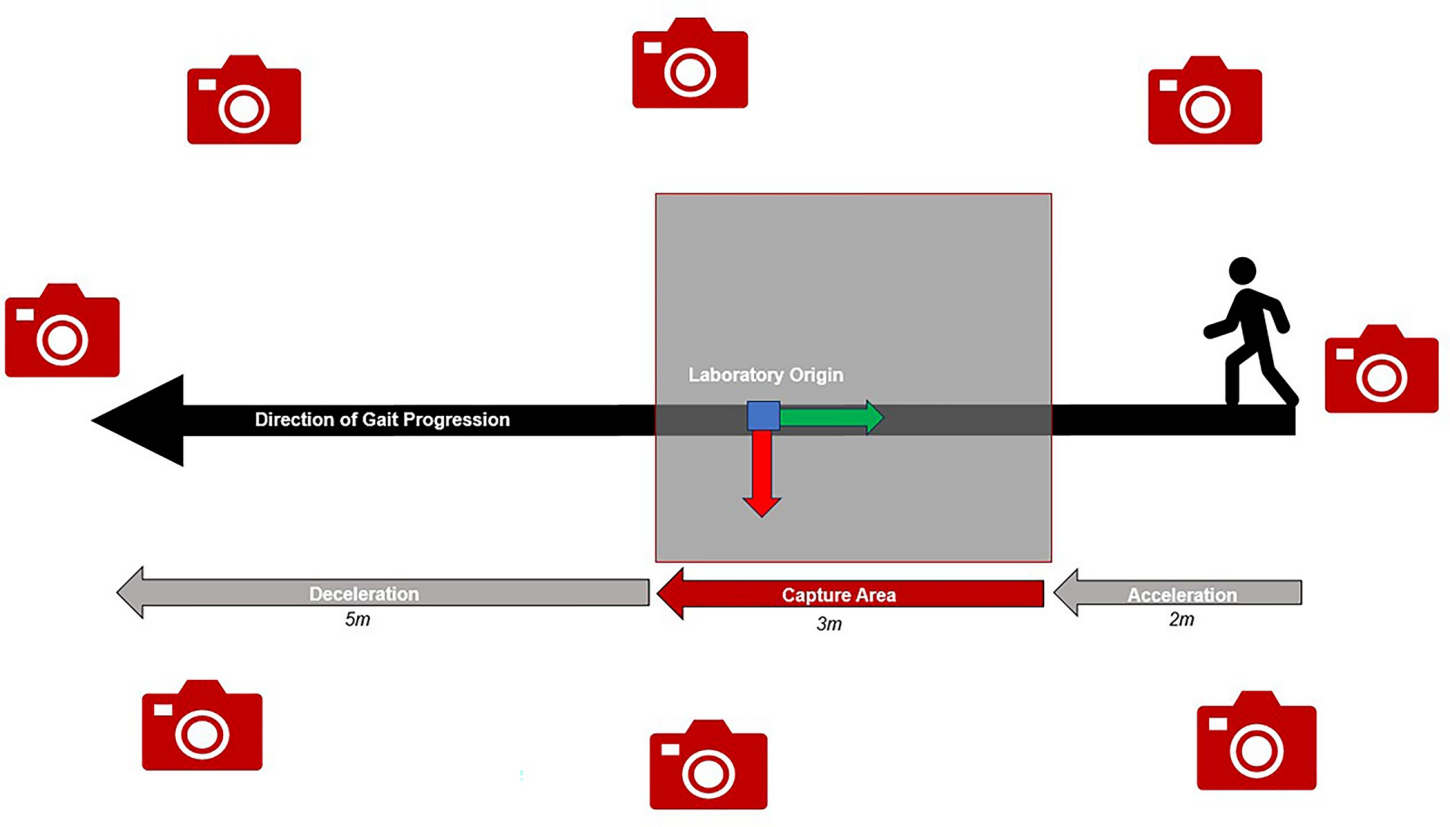
Example gait trial within 8-camera markerless motion capture laboratory. Gait trials comprised of a single gait cycle per limb progressed over an acceleration area of 2m, capture area of 3m, and deceleration area of 5m. Laboratory origin and axes are defined using the mediolateral (red), anteroposterior (green), vertical (blue) marks.

Pretest instructions to participants included cues to walk at their preferred pace, and to keep their gaze straight ahead. Following satisfactory completion of the practice trials used to ensure a full gait cycle was captured on each side (Fig 1), each subject completed five trials. Subjects were given a designated start position and then proceeded in a straight line at their preferred pace until traversing the entire walkway. If a trial failed to capture a full gait cycle, it was repeated until 10 successful trials were obtained. Ample rest (≈20s) was provided between trials.

### Data analysis

The keypoint estimation algorithm used the ResNet-152 backbone network to generate 2D spatial heatmaps for 40 keypoints representing body part and joint center locations. The adapted model was retrained using ground truth annotations from videos collected in the laboratory as well as an additional synthetic human image dataset using image augmentation techniques.

Initially, the algorithm used a bounding box image segmentation technique for person detection in the capture volume. This produces 3D spatial bounding boxes to determine the volume of the subject and reduce the area of the images processed by the keypoint estimation model. Bounding box results from the previous frame were input to the next frame of the image sequence to optimize processing. Next, the 2D segmented images from each camera view were processed using the keypoint estimation model yielding 2D coordinate estimates in each camera view. 3D coordinates were reconstructed by triangulating 2D keypoint estimates. This process was repeated across each frame of the image sequence. Segment pose estimations were obtained using adjacent keypoint estimates on each segment and are represented by a 4x4 transformation matrix containing the segment orientation and its position vector relative to the global frame. Segment poses were then input to Visual 3D (Visual 3D, HAS-Motion, Kingston, Ontario, Canada) for lower extremity kinematic modeling, gait event determination, and spatiotemporal parameter estimation.

The kinematics of interest were the three lower extremity joints (hip, knee, and ankle) and four lower extremity segments (pelvis, thigh, shank, and foot) for the left and right limbs in the sagittal, frontal, and transverse planes. Custom Visual 3D scripts were implemented to compute the lower extremity kinematics of interest from KinaTrax generated C3D data. Joint rotations were expressed as the distal segment relative to the proximal segment with the x-, y-, and z-axes representing sagittal, frontal, and transverse rotations. Y- and z-axis rotations were negated for the left lower extremity joints to maintain consistent interpretation with the right-hand rule. Segment rotations were expressed relative to the direction of gait progression with x-, y-, and z-axes representing sagittal, frontal, and transverse rotations. Similar to joint angles, y- and z-axes rotations were negated on the left side. Due to the exclusion of kinetic data, a constraint in many clinical settings, gait event determination was performed with a velocity-based method [29]. This is accomplished by using the positive zero-crossing of the anteroposterior velocity of the proximal foot segment relative to the pelvis segment to indicate heel-strike (HS) events, while the negative zero-crossing of the distal foot segment relative to the pelvis segment indicates toe-off (TO) events. Previously, this method has been applied during comparisons of gait analysis using KinaTrax in healthy adults (young and old) and adults with PD [14, 28]. Joint and segment kinematics were time normalized to 101 points using consecutive HS events to represent 0–100% of the gait cycle for each trial. Additional Visual 3D scripts were used to estimate spatiotemporal parameters of interest (Table 1). Following, both spatiotemporal parameters and time-normalized kinematics were exported from Visual 3D and combined for statistical analysis.

**Table 1:** Description of spatiotemporal parameters calculated from gait events in Visual 3D for both marker-based and markerless systems.

Gait Parameter	Description
Swing Time (s)	Period while the foot is not in contact with the ground (TO-HS in same leg).
Stance Time (s)	Period when the foot is in contact with the ground (HS-TO in same leg).
Double Support Time (s)	Initial and final part of stance phase when two feet are in contact with the ground (global time = initial + final double support).
Step Time (s)	Period taken for one step measured from HS of one foot to HS of the other foot.
Stride/Cycle Time (s)	Total amount of time for the cycle measured between successive HS in the same leg.
Cadence (strides/min)	Number of strides taken in a minute.
Stride Length (m)	Distance travelled during one stride (or cycle); Distance between two successive HS in the same leg.
Step Length (m)	Distance between corresponding HS in both legs; HS to HS in opposite leg.
Speed (m/s)	Distance covered per second; Calculated as the measured stride length divided by the measured stride time.
Stride Width (m)	Perpendicular distance (frontal plane) between the position of the proximal ipsilateral foot at ipsilateral HS to the position of the proximal end position of the foot at the next contralateral HS.

HS: Heel-strike, TO: Toe-off.

## Statistical analysis

Trial data are comprised of 135 captures across three sessions, in nine subjects, using five randomly sampled trials per visit. A single gait cycle was analyzed within each trial for joint and segment angles along with spatiotemporal parameters. Following the methods of previous work [15], trial data from the left and right sides were combined into one result for each kinematic variable and several spatiotemporal parameters.

Similarities in joint and segment kinematic waveforms were assessed using the coefficient of multiple determination (CMD) [30, 31] with effect sizes interpreted as small (CMD > 0.04), medium (CMD > 0.25), and large (CMD > 0.64) [32].

Within- and between-session analyses were performed using the two-way mixed effects intra-class correlation coefficient (ICC_3,k_) to assess agreement in spatiotemporal parameters [33–35]. ICC values less than 0.5 indicate poor reliability, values of 0.5–0.75 indicate moderate reliability, values of 0.75–0.9 indicate good reliability, and values greater than 0.9 indicate excellent reliability [33]. Standard error of measurement [36] and minimal detectable change (MDC) values were calculated for each spatiotemporal variable using methods described previously [37, 38]. All statistical analyses were carried out in Python (3.10.12).

## Results

### Joint angles

Results for within- and between-session reliability of joint angles for the sagittal (X), frontal (Y), and transverse (Z) planes using the CMD±SD are presented Table 2. The sagittal plane had the highest within-session reliability across all joints. At the hip joints, the mean CMDs in the sagittal plane were large, with values of 0.988±0.005, 0.992±0.003 and 0.968±0.010, at the hip, knee, and ankle joints, respectively. The mean CMDs of the frontal plane were medium to large ranging from 0.542±0.215 to 0.796±0.093 and the mean CMDs of the transverse plane ranged from small to large across all three joints.

**Table 2:** Within- and Between-session lower extremity joint kinematics.

Variable		Within-Session CMD			Between-Session CMD		
		Mean	SD	CI95	Mean	SD	CI95
Hip	Sagittal	0.988	0.005	[0.984, 0.991]	0.975	0.019	[0.961, 0.990]
	Frontal	0.796	0.093	[0.724, 0.867]	0.684	0.169	[0.554, 0.814]
	Transverse	0.803	0.136	[0.698, 0.907]	0.747	0.146	[0.634, 0.860]
Knee	Sagittal	0.992	0.003	[0.990, 0.994]	0.989	0.004	[0.985, 0.992]
	Frontal	0.542	0.215	[0.376, 0.707]	0.459	0.236	[0.278, 0.640]
	Transverse	0.126	0.201	[-0.028, 0.281]	0.159	0.155	[0.040, 0.277]
Ankle	Sagittal	0.968	0.010	[0.960, 0.975]	0.944	0.024	[0.926, 0.963]
	Frontal	0.594	0.110	[0.510, 0.679]	0.519	0.132	[0.417, 0.620]
	Transverse	0.037	0.147	[-0.076, 0.150]	0.076	0.097	[0.001, 0.151]

CMD: Coefficient of Multiple Determination. SD: Standard Deviation. CI95: 95% Confidence Interval.

Results for between-session reliability were comparable to within-session across all three planes, with the CMD of the hip (0.975±0.019), knee (0.989±0.004), and ankle (0.944±0.024) largest in the sagittal plane. Frontal plane CMDs were medium in the between-session analysis, ranging from 0.459±0.236 to 0.684±0.169 as transverse plane CMDs were similar to within-session results across all three joints.

### Segment angles

Results for within- and between-session reliability of segment angles are presented with the orthogonal convention used for joint angles and can be found in Table 3. Segment angle reliability was similar to joint angle reliability except for the pelvis segment, which was lower in the sagittal plane and frontal plane, but higher in the transverse plane. Within-session sagittal plane reliability was largest for the shank segments (0.997±0.002), followed by the foot (0.994±0.002), and thigh segments (0.992±0.003). Frontal plane reliability was medium in the pelvis segment and largest in the thigh segments ranging from 0.496±0.259 to 0.854±0.113 while within-session transverse plane reliability was small in the shank segments and large in the pelvis segment.

**Table 3:** Within- and between-session lower extremity segment kinematics.

Variable		Within-Session CMD			Between-Session CMD		
		Mean	SD	CI95	Mean	SD	CI95
Pelvis	Sagittal	0.647	0.106	[0.566, 0.728]	0.468	0.148	[0.355, 0.582]
	Frontal	0.496	0.259	[0.298, 0.695]	0.409	0.287	[0.188, 0.629]
	Transverse	0.915	0.040	[0.884, 0.946]	0.855	0.098	[0.780, 0.931]
Thigh	Sagittal	0.992	0.003	[0.990, 0.994]	0.988	0.006	[0.983, 0.993]
	Frontal	0.854	0.113	[0.767, 0.942]	0.792	0.163	[0.667, 0.917]
	Transverse	0.589	0.205	[0.432, 0.747]	0.504	0.235	[0.324, 0.685]
Shank	Sagittal	0.997	0.002	[0.995, 0.998]	0.996	0.002	[0.994, 0.997]
	Frontal	0.768	0.075	[0.711, 0.826]	0.729	0.074	[0.673, 0.786]
	Transverse	0.164	0.176	[0.029, 0.300]	0.194	0.156	[0.074, 0.315]
Foot	Sagittal	0.994	0.002	[0.992, 0.996]	0.990	0.005	[0.986, 0.994]
	Frontal	0.740	0.139	[0.634, 0.847]	0.674	0.164	[0.548, 0.801]
	Transverse	0.723	0.162	[0.599, 0.848]	0.691	0.165	[0.565, 0.818]

CMD: Coefficient of Multiple Determination. SD: Standard Deviation. CI95: 95% Confidence Interval.

Between-session reliability was highly similar to within-session reliability except for the pelvis with CMDs in the sagittal plane of 0.988±0.006, 0.996±0.002 to 0.990±0.005 at the thigh, shank, and foot, respectively. Frontal plane reliability was again medium for the pelvis segment (0.409±0.287) and large for the foot segments (0.792±0.163) with transverse plane reliability similarly small in the shank segments and large in the pelvis segment.

### Spatiotemporal parameters

Results for within- and between-session reliability of spatiotemporal parameters are reported for five bilateral and seven unilateral variables (Table 4). Within-session reliability for the bilateral parameters; cycle time, double support time, speed, stride length, and stride width demonstrated excellent agreement with the lowest ICCs seen in double support time (0.988) and stride width (0.989). These were accompanied by small SEMs (0.011 s; 0.006 m) below their MDCs (0.029 s; 0.017 m). Results for the unilateral parameters; stance time, step length, step time, cadence, stride length, strides per minute, and swing time, demonstrated excellent agreement in both limbs with the lowest ICCs appearing in left (0.983) and right (0.986) swing time. High unilateral ICCs for left and right swing time were complemented by low SEMs (0.007 s; 0.006 s) below their MDCs (0.020 s; 0.016 s), respectively.

**Table 4:** Mean, standard deviation, within- and between-session reliability spatiotemporal gait parameters.

	Total		Within-Session				Between-Session			
Variable	Mean	SD	SEM	MDC	ICC	ICC_CI95	SEM	MDC	ICC	ICC_CI95
Cycle Time (s)	1.125	0.073	0.015	0.041	0.991	[0.980, 1.000]	0.034	0.095	0.919	[0.750, 0.980]
Double Limb Support time (s)	0.372	0.047	0.011	0.029	0.988	[0.970, 1.000]	0.023	0.064	0.904	[0.700, 0.980]
Speed (m/s)	1.218	0.120	0.024	0.066	0.991	[0.980, 1.000]	0.058	0.160	0.913	[0.730, 0.980]
Stride Length (m)	1.366	0.115	0.020	0.055	0.994	[0.980, 1.000]	0.028	0.077	0.980	[0.940, 1.000]
Stride Width (m)	0.172	0.028	0.006	0.017	0.989	[0.970, 1.000]	0.008	0.022	0.971	[0.910, 0.990]
*Left Leg*										
Stance Time (s)	0.749	0.056	0.010	0.027	0.993	[0.980, 1.000]	0.028	0.079	0.901	[0.690, 0.980]
Step Length (m)	0.678	0.053	0.014	0.038	0.984	[0.960, 1.000]	0.016	0.044	0.965	[0.890, 0.990]
Step Time (s)	0.561	0.036	0.009	0.026	0.983	[0.960, 1.000]	0.018	0.050	0.896	[0.680, 0.970]
Steps Per Minute	107.493	7.085	1.831	5.075	0.983	[0.960, 1.000]	3.593	9.958	0.894	[0.670, 0.970]
Stride Length (m)	1.359	0.104	0.025	0.069	0.988	[0.970, 1.000]	0.030	0.083	0.972	[0.910, 0.990]
Strides Per Minute	53.693	3.558	0.658	1.825	0.992	[0.980, 1.000]	1.657	4.593	0.919	[0.750, 0.980]
Swing Time (s)	0.373	0.025	0.007	0.020	0.983	[0.950, 1.000]	0.009	0.024	0.959	[0.870, 0.990]
*Right Leg*										
Stance Time (s)	0.751	0.060	0.015	0.041	0.986	[0.960, 1.000]	0.028	0.079	0.913	[0.730, 0.980]
Step Length (m)	0.688	0.066	0.011	0.029	0.995	[0.990, 1.000]	0.016	0.045	0.980	[0.940, 1.000]
Step Time (s)	0.564	0.040	0.007	0.020	0.992	[0.980, 1.000]	0.017	0.046	0.936	[0.800, 0.980]
Steps Per Minute	106.861	7.601	1.351	3.744	0.993	[0.980, 1.000]	3.025	8.384	0.944	[0.820, 0.990]
Stride Length (m)	1.372	0.128	0.017	0.047	0.996	[0.990, 1.000]	0.029	0.080	0.983	[0.950, 1.000]
Strides Per Minute	53.441	3.588	0.813	2.252	0.989	[0.970, 1.000]	1.619	4.488	0.925	[0.770, 0.980]
Swing Time (s)	0.377	0.024	0.006	0.016	0.986	[0.960, 1.000]	0.007	0.019	0.968	[0.900, 0.990]

SEM: Standard Error Measurement. MDC: Minimal Detectable Change, ICC: Intraclass Correlation Coefficient, CI95: 95% Confidence Interval. SD: Standard Deviation. Means and SDs include all trials across all sessions. SEMs, MDCs, and ICCs include data from five trials per session.

Between-session reliability for the bilateral parameters demonstrated excellent agreement with the lowest ICCs seen in gait speed (0.913) and double support time (0.904). Paired with these were low SEMs (0.058 m/s; 0.023 s) well below their respective MDCs (0.160 m/s; 0.064 s). Results for the unilateral parameters demonstrated good to excellent agreement in both limbs with the lowest ICCs in left cadence (0.894) and right stance time (0.913). The SEMs for left cadence (3.593 steps/min) and right stance time (0.028 s) were smaller than their MDCs of 9.958 steps/min and 0.079 s, respectively.

## Discussion

This is the first study that aimed to examine the within- and between-session reliability of the KinaTrax markerless motion capture system for gait kinematic and spatiotemporal parameters in healthy adults. It was hypothesized that within- and between-session reliability of the MMC system would range from good (0.75–0.9) to excellent (>0.9) in the sagittal, frontal, and transverse planes for all gait kinematics, excluding the hip joint and pelvis segments, which would demonstrate moderate reliability (0.5–0.75) in the frontal and transverse planes. Additionally, within- and between-session reliability would be excellent (>0.9) for spatiotemporal parameters when using the MMC system. The principal hypothesis was supported across the sagittal plane measurements except for the pelvis segment, alternatively, at the knee and ankle joints the reliability of the data is less conclusive with CMD levels of small and medium in the transverse and frontal planes, respectively, therefore the principal hypothesis is rejected. These findings are potentially attributed to a lack of standardized attire or virtual model joint constraints. The secondary hypothesis had supporting evidence in the form of spatiotemporal parameters with excellent ICC values (>0.9) in 11 of the 13 parameters, however, between-session ICCs for Step Time (0.896) and Steps per Minute (0.894) require rejection of the secondary hypothesis. Potential limitations including sample size alone, may explain the rejection of the secondary hypothesis with ICCs bordering on excellent.

Highlighting the application of KinaTrax as a reliable gait analysis instrument, results were comparable to findings of a review that reported the reliability of gait kinematics using traditional marker-based systems [39]. The authors determined the median values for the sagittal, frontal, and transverse plane reliability >.8, excluding the pelvis segment, frontal plane reliability >.7, and transverse plane reliability to be < .7, excluding the pelvis. When comparing the within- and between-session reliability of the gait kinematics estimated by markerless motion capture, results were comparable to prior work [15] in which between-session variability was slightly higher than within-session variability. Typically, between-session reliability of marker-based motion capture is susceptible to human error from inconsistent marker placement [40], however, markerless systems are not compromised by this limitation. A potential source of greater between-session variation is the lack of standardization associated with clothing color and shape, which can influence keypoint detection in computer vision models [41] and should be considered in markerless gait analysis protocols. Using participants of similar demographics, Tamura et al. [42] assessed flat ground and treadmill sagittal plane trunk, hip, and knee gait kinematics using a Kinect v2 sensor while requiring subjects to wear tight-fitting clothing. The authors found that throughout the entire gait cycle, ICCs of the trunk, hip, and knee angle were <0.6, indicating that causes other than the clothing article, color, or shape limited the reliability of this system. With agreement meeting or exceeding these standards across all three planes, the KinaTrax MMC system may expand its scope to gait analysis with increased efficiency to marker-based approaches while matching their reliability [29] and that of other MMC systems [15].

It is important to note the primacy of the sagittal plane when diagnosing movement disorder and disease via clinical gait analysis. Most motion associated with human gait occurs in the sagittal plane, dictating that most gait function is assessed within that plane. Similar to our CMD results, Delval et al. [43] reported excellent mean ICC values for gait kinematics in the sagittal plane at the hip, knee, and ankle of healthy control and PD subjects. These results begin to demonstrate the efficacy of the KinaTrax markerless system in clinical gait analysis, given its high within- and between-session reliability in the sagittal plane of healthy adults.

Although results in the frontal and transverse planes were comparable to previous work on marker-based [29] and markerless [15] systems it is worth noting the need for improvement of any system to provide holistic motion analysis. Yet to be elucidated is the impact the number and location of keypoint definitions in the KinaTrax model could have on measurement agreement. Areas of improvement are highlighted with the lowest ICCs in the transverse plane for the knee joint (0.126), ankle joint (0.037), and shank segment (0.164) along with poor reliability in the frontal plane of the pelvis segment (<0.496). Provided most global movement is performed in the sagittal plane and the greatest ranges of motion expressed occur in the sagittal plane, the relatively small magnitude of motion in the frontal and transverse planes poses an issue to MMC systems. Should these keypoints be closely defined to one another, they become insensitive to translations and rotations similar to the sagittal plane, and thus more error prone. Additionally, a limited number of keypoints on any given segment will heavily reduce the sensitivity of measurements. This version of the KinaTrax model operates with keypoint definitions for transverse plane motions such as internal and external rotation of the lower extremities using a keypoint at the patella center, potentially explaining the lower agreement in the frontal and transverse planes.

The hypothesis that excellent within- and between-session agreement would be present for all spatiotemporal parameters was supported by the findings that of the five bilateral and seven unilateral parameters assessed during between-session analyses, only the ICCs for Left Step Time (0.896) and Left Cadence (0.894) did not support this hypothesis. When compared to spatiotemporal parameters assessed with marker-based methods [44], both within- and between-session reliability from our analysis met this standard with values >0.98 and 0.90, respectively. Additionally, the within- and between-session results across spatiotemporal parameters reported in our study such as gait speed, stride length, and stride width were comparable to SEMs results in stroke and PD patients using a marker-based motion capture [38], a pressure sensing walkway [45], and MDCs of pressure sensing insoles [46]. Overall, the findings from this work support the utility of the KinaTrax MMC system as a reliable gait analysis instrument in healthy adults.

Although this study supports the use of the KinaTrax MMC system to reliably analyze gait within the sagittal plane, several limitations should be considered. First, it must be determined whether testing constraints that seek to mimic typical daily conditions have an impact on reliability. The lack of standardization of clothing dimensions and colors, or movement speed, are potential limiting factors that could have contributed to the accuracy and reliability of the analysis. As previously mentioned, existing research has yet to evaluate the impact of clothing color and size on the KinaTrax machine learning model and thus provides another opportunity for future research. Additionally, a lack of constraint on gait direction due to the laboratory design could have impacted the quality of the data that the machine learning model uses for its estimations. Variations in gait direction along with a variety of populations such as aging individuals or movement impaired groups like injured athletes or adults with PD would serve as meaningful questions future research should entertain. Lastly, the model version employed for pose estimation of the sample dataset was constrained to 6DoF across each joint and segment, introducing a higher possibility of error compared to constrained models that limit the typical motion expected of a given joint or segment [16]. By pairing this question with the impact of keypoint definitions on frontal and transverse plane reliability, future work can capitalize on yet to be released iterations of this model and determine if new keypoints are incorporated and how they impact measurement reliability. Answering these research questions would help determine whether it is appropriate to utilize a 6DoF model or if additional constraints are still necessary.

## Conclusion

This study examined the within- and between-session reliability of gait kinematic and spatiotemporal components estimated from the KinaTrax markerless motion capture system across three sessions. Within sessions, lower extremity kinematics were most consistent in the sagittal plane, with the lower reliability values in the frontal and transverse planes attributed to model keypoint definitions and analysis philosophy. Similarly, trials across multiple sessions shared the same pattern of results as those seen in a single session. Due to the inclusion of only healthy adults, further investigation is necessary to determine if this within- and between-session reliability is present in gait analyses of other populations. Future work should seek to understand what impact clothing and other surface objects have on markerless human pose estimation. Additionally, improvements in machine learning model training and analysis philosophy should remain a collaborative process with developers. As it is incumbent on the biomechanics community to maintain the standard of motion capture systems, capable of robust and reliable results when implementing markerless motion capture. By examining a variety of testing methodologies, environmental settings, populations, and analysis methods will ensure AI-driven markerless motion capture presents a valid and reliable gait analysis tool vital to the determination and management of movement impairments in laboratory, clinical, and natural environments.

## Supporting information

S1 File Kinematic dataset. A composite of lower extremity kinematic data.
(JSON)

S2 File Sampled kinematics. Lower extremity kinematics from five sampled trials per subject per session utilized for statistical analysis.
(JSON)

S3 File Spatiotemporal dataset. A composite of spatiotemporal parameters calculated from lower extremity kinematics.
(XLSX)

S4 File Sampled spatiotemporal parameters. Calculated spatiotemporal parameters from five sampled trials per subject per session utilized for statistical analysis.
(XLSX)

S5 File Kinematics statistics. Statistics calculated from five randomly sampled gait trials per subject per session for three joints and four segments of the lower extremities in the sagittal, frontal, and transverse planes.
(XLSX)

S6 File Spatiotemporal statistics. Statistics calculated from five randomly sampled gait trials per subject per session for spatiotemporal parameters.
(XLSX)
